# Ripening-Associated Changes in Fatty Acid Composition and Nutritional Indices in Caciocavallo Silano PDO Cheese

**DOI:** 10.3390/foods14091566

**Published:** 2025-04-29

**Authors:** Giuseppe Tardiolo, Eleonora Di Salvo, Simona Tringali, Giovanni Bartolomeo, Claudia Genovese, Maria Elena Furfaro, Anna Maria Sutera, Antonino Nazareno Virga, Nicola Cicero, Alessandro Zumbo

**Affiliations:** 1 Department of Biomedical, Dental Sciences, Morphological and Functional Imaging, University of Messina, Via Consolare Valeria 1, 98125 Messina, Italy; gtardiolo@unime.it (G.T.); edisalvo@unime.it (E.D.S.); giovanni.bartolomeo@unime.it (G.B.); 2 Institute for Agriculture and Forestry Systems in the Mediterranean, National Research Council of Italy, Via Empedocle 58, 95128 Catania, Italy; simona.tringali@cnr.it (S.T.); claudia.genovese@cnr.it (C.G.); 3 Meat and Agribusiness Chain Research Consortium, Polo Universitario dell’Annunziata, Viale Giovanni Palatucci 13, 98168 Messina, Italy; mariaelena.furfaro@corfilcarni.it (M.E.F.); antonino.virga@corfilcarni.it (A.N.V.); 4 Department of Chemical, Biological, Pharmaceutical and Environmental Sciences, University of Messina, Viale Ferdinando Stagno d’Alcontres 31, 98166 Messina, Italy; asutera@unime.it; 5 Department of Veterinary Sciences, University of Messina, Viale Giovanni Palatucci 13, 98168 Messina, Italy; alessandro.zumbo@unime.it

**Keywords:** dairy chemistry, lipid profile, cheese maturation, protected designation of origin (PDO), nutritional quality

## Abstract

Caciocavallo Silano PDO is a traditional Italian stretched-curd cheese produced in southern Italy, subjected to a minimum ripening period of 30 days. The present study aimed to characterize the chemical composition and fatty acid (FA) profile at three ripening stages (up to 120 days). The proximate composition, FAs profile, and lactose content of cheese samples from three production batches, all made with Friesian cow milk, were analyzed. The results showed significant compositional changes during maturation. Moisture content decreased from 46.5% in 30 days to 33.0% in 120 days, with a corresponding increase in protein and fat content. Lactose content was below the limit of quantification (LOQ) at all ripening stages, confirming its natural depletion over time. The FA analysis revealed thirty-five different FAs, including fourteen saturated fatty acids (SFAs), nine monounsaturated fatty acids (MUFAs), and twelve polyunsaturated fatty acids (PUFAs). Among SFAs, palmitic and stearic acids were the most abundant, while lauric and myristic acids significantly increased with ripening. The sum of MUFAs showed a slight decrease, mainly due to the reduction in oleic acid, which dropped from 22.6% to 21.3% over maturation. Conversely, PUFAs exhibited a significant increase, particularly linoleic and α-linolenic acids, which are associated with positive health effects. In particular, an increase in PUFA composition and an optimal omega-6/omega-3 ratio could have a positive effect on health, with a consequent enhancement of cardiovascular function. The findings suggest that cheese maturation influences its nutritional and lipid profile, with potential implications for consumer health. Future research should assess how feeding strategies and production methods impact the FA composition of Caciocavallo Silano PDO.

## 1. Introduction

In the context of food production, traditional agri-food products are recognized as a pivotal component of European cultural heritage [1]. The connection between a region and its distinctive cultural identity and customs has emerged as a compelling factor influencing consumer choice, prompting a preference for traditional products [2]. A recent increase in demand for local and traditional foods has been observed, often perceived as being of superior quality [3], more sustainable [2], and having a strong cultural identity [4] compared to industrially produced alternatives. Italy holds the highest number of Protected Designation of Origin (PDO), Protected Geographical Indication (PGI), and Traditional Speciality Guaranteed (TSG) products in Europe, with 56 varieties of cheese falling under these categories [5] (ISMEA Report). In 1999, Italy’s Ministry of Agriculture, Food Sovereignty, and Forests (MASAF) officially defined traditional agri-food products through Decree No. 350 (issued 08/09/99) [6]. The decree describes these products as being “obtained with processing methods, storage and maturation over time consolidated, homogeneous throughout the territory concerned, according to traditional rules, for a period not less than twenty-five years” [5]. The southern Italian dairy sector is renowned for its variety of fresh and aged stretched-curd (pasta filata) cheeses. The term “Caciocavallo” is traditionally used to refer to a semi-hard pasta filata cheese that has been historically produced in various regions of Italy [6], while the term “Silano” originates from the Sila mountain range located in the Calabria region [7]. Caciocavallo Silano is recognized with a Protected Designation of Origin (PDO) label under European Regulation 1236/96. The production of the cheese is prevalent in five regions of southern Italy: Campania, Basilicata, Calabria, Puglia, and Molise [8]. The cheese is crafted using whole cow’s milk, either raw or mildly heat-treated (58 °C for 30 s), with rennet added at a temperature of 36–38 °C. Fermentation lasts between 4 and 10 h, initiated by the addition of natural whey cultures obtained from previous cheese production following the traditional back-slopping method. The fermentation process is stopped based on empirical observation, once the curd is ready for stretching in hot water. After the stretching phase, the cheese is formed into flask-like shapes, typically weighing between 1 and 2.5 kg, and then cooled in water, salted in brine for 6 h, and hung to air-dry. The minimum ripening period is 30 days, but it may last up to 1 year and even longer, according to the cheese factory production procedures. In addition to cheesemaking factory procedures, animal breeding management can influence the composition of fatty acids (FAs) in dairy products, altering the FA profile [9]. However, Caciocavallo Silano producers must adhere to the PDO regulations (DPCM 196/93, Rome, Italy), which govern aspects such as milk source, processing, stretching method, and aging time [8]. As the chemical composition of milk can be influenced by the breed, effects can also be observed on the texture of cheese and its volatile composition, which may have implications for the sensory properties of the product [9]. The process of ripening in pasta filata cheese is characterized by the accumulation of microbial metabolism products, which have a significant impact on the sensory properties of the final product. This phenomenon is accompanied by substantial proteolysis, a result of bacterial activity [10]. Caciocavallo Silano PDO is also appreciated for its evolving sensory characteristics, such as texture, aroma, and flavor intensity. These characteristics develop progressively with ripening and influence consumer perception and acceptance. The milk used for its production often comes from Friesian cattle, a breed widely recognized for its high milk yield and adaptability to different management systems [11]. Moreover, Italian Friesian cows (Holstein–Friesian) are the dominant dairy breed in Italy, representing a key component of intensive dairy farming due to their superior milk productivity and composition, particularly in terms of fat and protein content [12]. The feeding regimen of dairy cattle plays a key role in shaping the compositional characteristics of milk and, consequently, the quality of dairy products. One of the most commonly adopted feeding systems in modern dairy farms is the unifeed diet, also known as total mixed ration (TMR). This system ensures a balanced and homogeneous intake of nutrients by mixing forages and supplements into a single ration, optimizing both rumen function and milk composition [13]. The unifeed diet not only improves feed efficiency but also influences the FA profile of milk, particularly enhancing the levels of monounsaturated fatty acids (MUFAs) and polyunsaturated fatty acids (PUFAs), which are crucial for human health [14]. Given the importance of the Friesian breed and the impact of the unifeed system on milk quality, understanding their influence on Caciocavallo Silano PDO’s composition is essential. Numerous studies have examined the microbial and biochemical properties of Caciocavallo cheeses from southern Italy, identifying significant variations among seemingly similar products [15,16,17]. In this context, it is evident that variations in the provenance of cheese originating from PDO regions and non-PDO regions hold considerable promise for practical applications in the context of safeguarding the authentication and tracking the provenance of protected status cheeses. Consequently, the present study has selected Caciocavallo Silano DOP PDO as the authentic Italian cheese, with the objective of characterizing the chemical composition and FA profile of Caciocavallo produced in the Calabria region at three stages of ripening: 30, 60, and 120 days. This traditional cheese embodies the region’s cultural heritage and reflects the expertise passed down through generations.

## 2. Materials and Methods

### 2.1. Farm Management and Sample Collection

Cheese samples were collected from a dairy farm located in the province of Cosenza, Calabria, southern Italy (39.2982° N, 16.2536° E). A total of five Caciocavallo Silano PDO samples were collected at three maturation stages (30, 60, and 120 days), originating from three distinct production batches, and analyzed in triplicate. Each sample weighed between 250 and 300 g. All cheeses were ripened in a traditional aging cellar at 10 ± 1 °C and 85–90% relative humidity, under PDO-standard conditions. The milk used for cheesemaking was sourced from the farm’s Friesian cattle herd, which consists of 150 animals. The herd is housed in a free-stall system with unrestricted access to water. Cows were fed a TMR diet comprising 35% maize and 15% barley as cereals, 12% soybean meal, 5% sunflower meal, 8% maize gluten feed as protein sources, and 25% polyphyte hay from organic farming. Barn conditions were optimized for milk production, with an average temperature of 20 ± 2 °C and a relative humidity of 65%. Milk from the three production batches had an average protein content of 3.21 g/100 g, lactose content of 4.55 g/100 g, and fat content of 3.85 g/100 g. These values were derived from routine quality control procedures carried out by the dairy.

### 2.2. Chemical Composition of Feed and Cheese

Representative samples were homogenized and analyzed in triplicate. Moisture was determined following the AOAC International, 2000, method 948.12 for cheese and 930.15 for unifeed [18]. Ash was determined following the AOAC International, 2000, method 935.42 for cheese and 942.05 for unifeed [19,20]. Protein was determined by the Kjeldahl method (unifeed by ISO 1871:2009 method and cheese by AOAC International, 2000, method 920.123) [21]. Fat was determined following the AOAC International, 2000, method 933.05 for cheese and method 920.39 for unifeed [22,23]. Crude fiber in unifeed was determined by the Weende method (AOAC International, 1996, method 978.10) [24]. In addition, the Van Soest method was used for fiber analysis, which differentiates the fiber into three fractions depending on how it reacts to various types of solvent: NDF (Neutral Detergent Fiber) (AOAC International, 2005, method 2002.04), ADF (Acid Detergent Fiber) and ADL (Acid Detergent Lignin) (AOAC International, 1977, method 973.18) [25]. NDF is a good indicator of “bulk” and thus feed intake. ADF and ADL are good indicators of digestibility and thus energy intake.

### 2.3. Salt Content in Cheese

Sodium content was analyzed by inductively coupled plasma mass spectrometry (ICP-MS) after microwave-assisted acid digestion. Digestion conditions for the microwave system applied were as follows: up to 180 °C for 20 min and then constant for 30 min; finally, a cooling stage (30 min) was carried out to 22 °C, and samples were diluted to 50 mL with deionized ultrapure water. This solution was filtered with membrane filters (0.45 µm) and performed with an ICP-MS equipped with a concentric Nebulizer, a quartz torch with quartz injector tube, and cyclonic spray chamber. The concentration of sodium was determined. External calibration curves from standard solutions were used to quantify the amount of sodium. Salt content was calculated using the following formula: salt = sodium × 2.5 according to the Annex A of the Regulation (Eu) No 1169/2011 of the European Parliament and of the Council of 25 October 2011 [26].

### 2.4. Lactose Analysis in Cheese

Samples were homogenized with 25 mL H_2_O and incubated at 70 °C in a shaking water bath for 20 min. They were clarified with Carrez solutions (Carrez I and Carrez II) and diluted 1:10. Samples were cooled to 2 °C for 20 min and then filtered with membrane filters (0.45 µm). The lactose content in cheese was determined using an HPLC Shimadzu Nexera XR with refractive index detection. Column temperature was maintained at 35 °C, the injection volume was 20 μL, and the flow rate was 1 mL/min. The eluent consisted of acetonitrile/water 60:40 (*v*:*v*). Lactose was identified by comparing the retention time to that of a reference standard. An external calibration curve produced by standard solutions was used to quantify the amount of lactose in each sample. The limit of detection (LOD) for the calibration curve of lactose analysis was determined to be 0.05 g/100 g, while the limit of quantification (LOQ) was established at 0.1 g/100 g.

### 2.5. Fatty Acids Analysis in Unifeed and Cheese

Lipid fraction extraction was carried out according to Folch et al. [27], with slight modifications. Four grams of finely ground sample were transferred into 50 mL tubes, added to 40 mL of a mixture of chloroform and methanol (2:1, *v*/*v*), and extracted by an ultrasonic bath (FALC LBS1) for 30 min. Subsequently, 10 mL of a 0.73% aqueous sodium chloride solution was added. The samples were vortexed for 1–2 min and then centrifuged (Awel MF 20-R) at 7000 rpm for 15 min. The lipid fraction (bottom layer) was collected in a preweighed flask and dried using a rotating evaporator (BÜCHI, Rotavapor R-210). The total lipid content (g/100 g) was determined through gravimetry. FA methyl esters (FAMEs) were obtained by trans-methylation of the lipid fractions of the analyzed samples. This process was performed by adding a methylating mixture consisting of methanol and sulfuric acid (9:1, *v*/*v*) at equal parts of lipid extract (ISO 5509:2000 method) [28]. Subsequently, the mixture was placed in an oven at 100 °C for 1 h, with butylated hydroxytoluene (BHT) serving as an antioxidant to protect polyunsaturated FAs (PUFAs) from high temperatures. The supernatant was then diluted with *n-*hexane (1:10) and analyzed. The FA composition of the samples was determined by a gas chromatograph (Master GC-DANI), equipped with a split/splitless injector, a capillary column (Phenomenex ZB-Wax, 30 m × 0.25 mm, film thickness 0.25 µm), and a flame ionization detector FID [29,30]. The modifications applied to the Folch method were based on protocols previously validated for cheese matrices, optimizing lipid recovery and minimizing degradation during extraction [27]. The analyses were carried out under the following instrumental conditions: column temperature ranged from 50 °C (2 min) to 240 °C (15 min) with a heating rate of 3 °C/min; injector and FID temperatures were set at 240 °C; carrier gas helium was used flowing at a constant linear velocity of 30 cm/s; injection volume was 1 µL, with a split ratio of 1:50. All determinations were performed in triplicate. Data acquisition and management were performed using Clarity Chromatography software (version 4.0.2). The FAMEs were identified by comparing their retention times with reference compounds present in two mixtures (Supelco 37-component FAME mix and Supelco Mehaden oil).

### 2.6. Atherogenicity Index (AI) and Thrombogenicity Index (TI)

The atherogenic index (AI) and thrombogenic index (TI) are indicators used to evaluate the potential cardiovascular impact of FA profiles, with lower values generally considered more favorable for health. The AI and the TI were measured to evaluate and provide data on the health and nutritional value of the lipids in the Caciocavallo cheese samples evaluated [31]. The AI was calculated as the ratio between the sum of the main saturated FAs (SFAs) and the sum of the main classes of unsaturated FAs. The AI is the ratio of saturated to unsaturated FAs. SFAs are pro-atherogenic, while unsaturated FAs are anti-atherogenic. This may reduce the risk of coronary diseases [32]. The following equation was used to calculate the AI:

AI = [C12:0 + (4 × C14:0) + C16:0]/[Σ n-6 PUFA + Σ MUFA + Σ n-3 PUFA]

The TI is a measure of blood clotting. The method of calculation involves a comparison of prothrombogenic saturated FA (SFAs) and antithrombogenic saturated FA (MUFAs, n-6 PUFA, n-3 PUFA) [33,34]. The TI was calculated using the following equation:

TI = [C14:0 + C16:0 + C18:0]/[0.5 × Σ n-6 PUFA + 0.5 × Σ MUFA + 3 × Σ n-3 PUFA + (n-3 PUFA/n-6 PUFA)]

### 2.7. Statistical Analysis

Differences in the contents of FAs (SFAs, MUFAs, and PUFAs) during cheese ripening at 30, 60, and 120 days were subjected to Bartlett’s test for homogeneity of variance and then analyzed using factorial analysis of variance (ANOVA), using CoStat software version 6.451 (CoHort software, Montenery, CA, USA). The dataset was processed using the D’Agostino–Pearson K2 test for normality and the Student–Newmann–Keuls test for multiple comparisons. Significance was set at the *p* ≤ 0.05 level (Snedecor and Cochran 1989) [32]. FA composition scores were used to construct a principal component analysis (PCA) biplot in R (version 3.5.3).

## 3. Results

### 3.1. Chemical Composition of Feed

In this study, the chemical composition of the unifeed administered to the dairy cows was analyzed to better understand its influence on milk fat composition and, consequently, on cheese lipid profile. As shown in Table 1, the unifeed contained 12.4% protein, 6.2% fiber, and 4% fat, alongside reduced levels of ADF and ADL, which are known to affect digestibility and energy availability. In summary, the most abundant FAs in the unifeed were palmitic acid (C16:0), oleic acid (C18:1 n-9), and linoleic acid (C18:2 cis), with linoleic acid representing nearly 48% of the total. These dietary FAs are partially transferred to milk through ruminant digestion and biohydrogenation processes, contributing significantly to the cheese’s final FAs composition.

In summary, the most abundant FAs identified in unifeed were palmitic acid (C16:0), oleic acid (C18:1 n-9), and linoleic acid (C18:2 cis), with linoleic acid showing a substantial concentration (47.67%). However, relatively low concentrations of MUFAs and PUFAs were observed. Data concerning the FAs profile are reported in Table 2.

### 3.2. Chemical Composition of Cheese

In the case of cheese, significant variations in chemical composition are evident among different ripening stages. The chemical composition of Caciocavallo Silano is shown in Table 3. The ripening time had a significant effect on ash and protein, but the most substantial changes were observed in moisture (from 46.55% to 32.0%) and salt (from 1.96% to 1.17%) composition. As moisture decreased, salt concentration increased. No variations in the lactose parameter were observed as a consequence of the ripening time. In accordance with Italian legislation, the designation “lactose-free” may be employed for dairy products containing a lactose residue of less than 0.1 g per 100 g. However, the feeding strategy also contributed positively to the cheese’s chemical profile. The lactose content remained below the limit of quantification (LOQ = 0.05 g/100 g) across all stages, qualifying the product as “lactose-free” according to Regulation (EU) No. 1169/2011 [26]. This is likely due to the enzymatic activity of lactic acid bacteria, which degrade residual lactose during early ripening [35].

### 3.3. Fatty Acid Profile of Cheese

In Caciocavallo Silano PDO cheese, thirty-five FAs from C4:0 to C24:1 n-9 were identified (Table 4). In particular, fourteen SFAs, nine MUFAs, and twelve PUFAs. Among the SFAs identified, eleven showed significant variations, of which six (C8:0, C12:0, C17:0, C20:0, C22:0, and C24:0) showed an increase during maturation, while the most abundant SFAs, palmitic (C16:0) and stearic acids (C18:0), showed a slight decrease during maturation.

**Figure 1:**
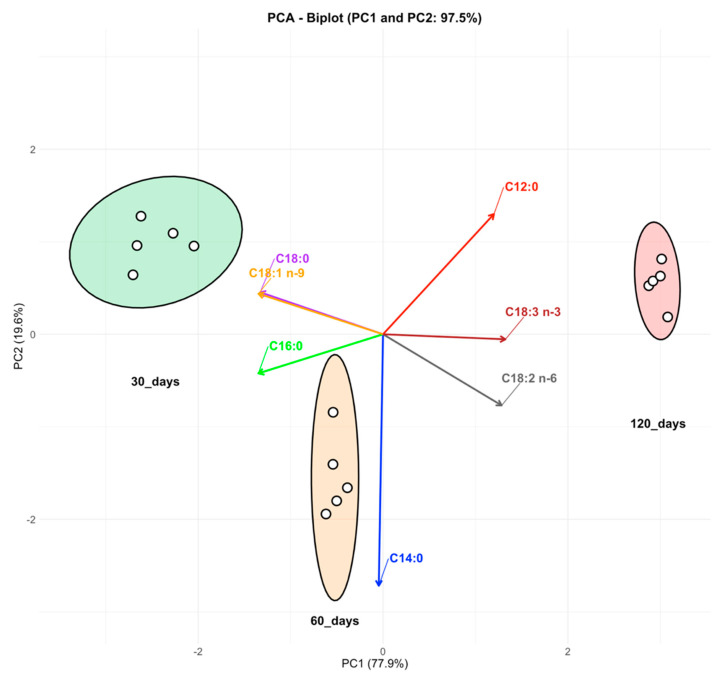
Biplot of principal component analysis (PCA) based on the relative content of selected fatty acids (C12:0 red, C14:0 blue, C16:0 green, C18:0 purple, C18:1 n-9 orange, C18:2 n-6 grey, and C18:3 n-3 brown) in Caciocavallo Silano PDO at three ripening stages (30, 60, and 120 days). The ellipses indicate the clustering of samples at each ripening stage. Arrows represent the most health-relevant fatty acids, with their direction and magnitude indicating their contribution to the principal components. The first two principal components explain 97.5% of the total variance.

Among the SFAs, C12:0, C14:0, and C16:0 were identified as pro-atherogenic, suggesting that a modest decline in C16:0 during maturation may have a positive effect on this index, whereas C18:0 is converted to oleic acid (C18:1 n-9) in human tissues and has a hypocholesterolemic effect [36]. The sum of the SFAs did not demonstrate significant variations during the maturation process. As regards the MUFAs, five showed significant differences and four of them increased during ripening, namely myristoleic acid (C14:1), trans-palmitoleic acid (C16:1 n-7), heptadecanoic acid (C17:1), and eicosenoic acid (C20:1 n-9), while oleic acid (C18:1 n-9), the most abundant among the MUFAs, showed a slight decrease during ripening. The sum of MUFAs demonstrated a significant decrease during the ripening process of Caciocavallo Silano, from 26.0% (30 days) to 25.3% (120 days). This decline can be primarily attributed to the decrease in oleic acid (C18:1 n-9), which alone accounts for approximately 84% of total MUFAs. This decline has a substantial impact on the overall result. Furthermore, to investigate the compositional changes in the most health-relevant FAs during the ripening process of Caciocavallo cheese, PCA was performed on a selected group of FAs (C12:0, C14:0, C16:0, C18:0, C18:1 n-9, C18:2 n-6, and C18:3 n-3). The first two principal components (PC1 and PC2) collectively account for 97.5% of the total variance, enabling a clear differentiation between the three ripening stages (Figure 1). The PCA biplot reveals distinct clustering of samples, with well-defined ellipses representing the 30-day, 60-day, and 120-day ripening stages, indicating a gradual shift in FA composition during ripening. The distribution of fatty acids along PC1 and PC2 indicates that shorter-chain SFAs (C12:0, C14:0, and C16:0) are found to be significant contributors to PC1, primarily affecting the separation of the 30-day samples. Stearic acid (C18:0) and oleic acid (C18:1 n-9) exhibited a tendency to cluster near the 60-day samples, suggesting a shift in the balance of saturated and monounsaturated FAs. The PUFAs, C18:2 n-6 and C18:3 n-3, exhibited a shift towards the 120-day samples, indicating an increase or metabolic transformation over time. These results underscore the substantial impact of ripening on the fatty acid composition of Caciocavallo, with potential implications for its nutritional quality and lipid profile.

## 4. Discussion

Several studies have demonstrated that both the proximate composition and the FAs profile of cheese can be significantly influenced by multiple factors, including breed, feeding strategy, seasonal production, and the chemical characteristics of the raw milk used [37,38,39]. In this study, ash, protein, salt, and fat content progressively increased during maturation, while moisture gradually declined. In particular, at the end of ripening, protein and fat composition changed the most, in disagreement with the results reported by Ianni et al. [40], where the ripening time did not influence the proximate composition. Regarding the effect of cheese ripening time (Table 3), a significant time trend was noted for all cheese parameters. However, between 30 and 120 days of ripening, there was an expected decrease in water concentration (moisture) and an increase in salt content. Total salt content was positively correlated with moisture, protein, and total FAs content, in agreement with the findings of Fallico et al. [41] on the ripening of Ragusano cheese, and with Panari et al. [42] on Parmigiano Reggiano cheese. Moreover, the observed reciprocal trend between salt and moisture likely reflects passive salt diffusion into the matrix as water content decreases, a phenomenon previously reported in other ripened cheeses [37,38]. On the other hand, the FAs profile (Table 4) of cheese fat was mainly influenced by feed and nutrition, while ripening time showed no relevant changes. Research conducted on a range of cheeses has consistently demonstrated that the application of technology and the duration of ripening do not significantly influence the FAs composition of the cheese, irrespective of its type [37]. The present study corroborated the hypothesis that the Caciocavallo Silano is naturally devoid of lactose, as previously proposed by Di Trana et al. [43]. In addition, the analysis revealed that this cheese is abundant in bioactive compounds, including branched-chain fatty acids, vitamins A and E, and polyphenols, when compared with other cheeses with similar fat content. This traditional cheese product has been found to contain a high nutritional value, with a low PUFA-n6/n3 ratio. In accordance with nutritional recommendations, this links it to the category of excellent functional nutrition. In relation to the FAs composition, the oleic acid (C18:1 n-9) content observed in Caciocavallo Silano at 120 days of ripening may reflect the dietary regimen adopted in the farm, based on unifeed feeding with hay and cereals, as also observed by Esposito et al. [44]. This is in line with the composition of the unifeed used in this study (Table 2), which contained about 31% oleic acid. As widely documented, dietary FAs are partially transferred from feed to milk through the digestion and metabolism processes in ruminants [45]. Despite the biohydrogenation that occurs in the rumen—which converts a portion of unsaturated FAs into saturated ones—a significant fraction of dietary MUFAs such as oleic acid escapes this process and reaches the mammary gland, ultimately contributing to the lipid profile of milk and, consequently, of the derived cheese [9,45]. Several studies have shown that including oilseeds (e.g., flaxseed, rapeseed) or vegetable oils (e.g., soybean, sunflower oil) in the diet can enhance the concentration of MUFAs and PUFAs in milk, contributing to an improved nutritional quality of dairy products [46,47]. From a nutritional perspective, the intake of MUFAs such as oleic acid and PUFAs like α-linolenic acid (ALA) and conjugated linoleic acid has been associated with potential positive effects on cardiovascular health, modulation of inflammatory responses, and improved metabolic control in type 2 diabetes [48,49,50]. In this context, the cheese analyzed in this study appears to reflect the dietary origin of the milk and its influence on the final lipid composition of the product. The twelve PUFAs identified in Caciocavallo Silano mostly belong to the essential omega-6 and omega-3 FA families. These essential FAs cannot be synthesized by the human body and must therefore be obtained from the diet. Their importance lies in their recognized health-promoting functions, particularly in cardiovascular protection, the modulation of inflammation, and the support of immune responses [51,52]. The levels of linoleic acid (LA, C18:2 n-6) and ALA (C18:3 n-3) detected in the samples are consistent with those typically found in cheeses made from the milk of cows raised indoors and fed on conserved forages and cereals [38]. LA, a representative of the omega-6 series, is involved in various physiological functions including skin integrity, cell membrane structure, and eicosanoid synthesis. ALA, part of the omega-3 family, plays a role in the prevention of chronic inflammatory diseases and contributes to maintaining vascular homeostasis due to its anti-inflammatory and antioxidant activity [53,54].

Despite concerns about the imbalance between omega-6 and omega-3 FA intake, the ratio recorded in this study (4.6 at both 30 and 120 days of ripening) falls within the range considered beneficial for human health [55,56]. In addition, the omega-6/omega-3 ratio of 4.6 is well below the upper threshold of 5:1–6:1 recommended by EFSA and WHO for cardiovascular risk reduction, thus supporting the favorable nutritional profile of the product [57]. This ratio is in line with the recommendations for cardiovascular protection and supports the antithrombogenic profile of the cheese fat [26,43,51,52]. Moreover, the overall PUFA content showed a significant increase during maturation, rising from 3.4% at 30 days to 4.0% at 120 days. Although modest, this trend may reflect both lipolytic processes occurring during ripening and the possible release or redistribution of certain lipid fractions over time [58]. Lipolytic changes observed may be linked to enzymatic activity by indigenous or non-starter lactic acid bacteria, which are known to produce lipases capable of hydrolyzing milk fat and releasing PUFAs over time [59,60]. As regards the statistical results, a notable trend observed is the shift in C18:2 n-6 and C18:3 n-3 over time, suggesting that the oxidation or metabolic conversion of these FAs may contribute to the observed differences in the ripening stages. Similarly, the high contribution of C12:0 and C14:0 to PCA suggests that their levels are critical factors distinguishing early-stage ripening samples from those at 120 days. The divergent trends in individual SFA concentrations may reflect specific lipolytic activities occurring during ripening, which selectively hydrolyze triglycerides containing medium-chain FAs (e.g., C12:0, C14:0), while longer-chain SFAs like C16:0 and C18:0 may be further metabolized or redistributed within the matrix. The PCA revealed that early-stage ripening samples were clustered based on higher levels of short-chain SFAs, while later stages were associated with increased PUFAs content. These differences underscore the progressive lipid remodeling during cheese maturation. These results highlight how the ripening process influences the lipid composition of Caciocavallo, potentially affecting its nutritional profile and health properties. These findings are consistent with those of previous studies, which reported alterations in the FAs composition of cheese during the ripening process [59]. Furthermore, the variation in the AI and TI observed during ripening aligns with previous reports on aged cheeses, where microbial metabolism and enzymatic lipolysis were shown to modify the lipid composition over time [56]. These processes, primarily driven by the activity of endogenous enzymes and specific microbial populations, may selectively alter the proportion of individual FAs, potentially increasing the relative presence of SFAs during prolonged maturation. Such transformations are not only technologically relevant but also nutritionally significant, as they can affect lipid quality indices [56]. Additionally, other studies have reported that the TI and AI values of goat cheese are generally lower than those of other types of cheese [60]. However, the data reported indicated levels that were higher than those typically observed in other types of cheese derived from sheep and goat milk. This finding suggests that Caciocavallo Silano cheese possesses a diverse profile of FAs and may offer distinct health benefits. The variations in the composition of FAs and the divergent values of lipid quality indices in the cheeses may be ascribed to the disparities in the composition of the milk utilized in their production and the technology employed in the cheesemaking process.

Future investigations should explore how variables such as temperature and humidity, with microbial analysis and sensory evaluations, during cheese aging influence the AI and TI values, offering new insights into the formulation of strategies aimed at enhancing the nutritional profile of traditional dairy products like Caciocavallo Silano PDO. It is imperative to acknowledge the limitations of this study when interpreting the findings. Firstly, the sample size was restricted to three production batches under controlled conditions, which may restrict the generalizability of the results. Secondly, the absence of microbiological and sensory evaluations has constrained the interpretation of compositional changes in relation to flavor or microbial activity. Finally, the minimization of environmental variability during the aging process was beneficial for standardization purposes. However, it should be noted that this may not reflect real-world variability across producers.

## 5. Conclusions

This study offers a comprehensive characterization of the chemical composition and FAs profile of Caciocavallo Silano PDO across three stages of maturation. The results confirm that ripening significantly affects moisture, protein, fat, and salt contents, with a gradual moisture loss and a corresponding concentration of dry matter components. Maturation also led to notable changes in the FAs composition, particularly a reduction in palmitic (C16:0) and LA (C18:1 n-9), alongside an increase in lauric (C12:0), myristic (C14:0), and PUFAs such as LA (C18:2 n-6) and ALA (C18:3 n-3). These variations may enhance the nutritional value of the cheese, especially in light of the favorable omega-6 to omega-3 ratio observed. The PCA analysis supported these compositional shifts, highlighting clear separation between ripening stages and underscoring the evolving lipid profile. Overall, the results suggest that cheese maturation not only affects technological properties but may also shape its nutritional profile in a health-relevant manner. These findings may guide producers in selecting optimal ripening durations to enhance the nutritional profile of traditional cheeses. Furthermore, the optimization of ripening conditions may prove beneficial in increasing PUFAs content. Future research should investigate the influence of alternative feeding strategies (e.g., pasture-based vs. unifeed systems) and microbial cultures on the lipid and sensory properties of Caciocavallo Silano PDO. Such insights could support the development of production practices that enhance nutritional quality while preserving the unique features guaranteed by its PDO status.

## Figures and Tables

**Table 1:** Nutritional composition of unifeed.

Unifeed	%
Moisture	8.8
Ash	5.3
Protein	12.4
Fat	4.0
Fiber	6.22
NFE	63.28

The results are expressed as composition percentage. NFE = N-free extracts.

**Table 2:** Fatty acid expressed in % of total FAMEs identified in unifeed.

Fatty Acid	Unifeed (%)
C8:0	1.19
C10:0	2.99
C11:0	0.05
C12:0	0.06
C13:0	0.03
C14:0	0.11
C15:0	0.02
C15:1	0.01
C16:0	12.54
C16:1 n9	0.04
C16:1 n7	0.08
C17:0	0.09
C17:1	0.03
C18:0	2.95
C18:1 n9	31.41
C18:1 n7	0.82
C18:2	47.67
C18:3 n6	0.03
C18:3 n3	1.74
C20:0	0.64
C20:1 n11	0.07
C20:1 n9	0.43
C20:2 n6	0.04
C21:0	0.03
C20:4 n3	0.02
C22:0	0.40
C22:1 n11	0.01
C22:1 n9	0.07
C23:0	0.08
C24:0	0.38
C24:1	0.06

The results are expressed as composition percentage.

**Table 3:** Chemical composition of Caciocavallo Silano cheese expressed in g/100 g of cheese ± standard deviation.

Caciocavallo Silano	Moisture	Ash	Protein	Fat	Salt	Lactose
30 days	46.55 ± 0.19	2.82 ± 0.01	24.52 ± 0.81	24.94 ± 0.04	1.17 ± 0.14	<LOQ
60 days	37.20 ± 0.17	3.53 ± 0.04	29.08 ± 0.74	28.6 ± 0.13	1.59 ± 0.25	<LOQ
120 days	32.0 ± 0.09	4.54 ± 0.18	31.5 ± 0.86	30.0 ± 0.07	1.96 ± 0.20	<LOQ

Data reported as means ± standard deviation. The limit of quantification (LOQ) = 0.05%.

**Table 4:** Fatty acid expressed in % of total FAMEs identified ± standard deviation in “Caciocavallo Silano” cheese at 30, 60, and 120 days of ripening.

		Ripening		
Fatty Acid	30 Days	60 Days	120 Days	Significance
C4:0	2.94 ± 0.25	2.97 ± 0.21	3.05 ± 0.16	ns
C6:0	2.29 ± 0.09	2.60 ± 0.06	2.55 ± 0.53	ns
C8:0	1.29 ± 0.05 ^c^	1.61 ± 0.06 ^b^	1.98 ± 0.05 ^a^	***
C10:0	3.18 ± 0.04 ^a^	2.45 ± 0.02 ^c^	2.77 ± 0.05 ^b^	***
C12:0	3.30 ± 0.04 ^b^	3.19 ± 0.04 ^c^	3.93 ± 0.02 ^a^	***
C13:0	0.06 ± 0.03	0.08 ± 0.04	0.10 ± 0.04	ns
C14:0	10.90 ± 0.02 ^b^	11.10 ± 0.05 ^a^	10.93 ± 0.02 ^b^	***
C15:0	1.09 ± 0.11 ^ab^	1.15 ± 0.13 ^a^	0.93 ± 0.13 ^b^	*
C16:0	32.72 ± 0.02 ^a^	32.50 ± 0.02 ^b^	31.69 ± 0.02 ^c^	***
C16:1 n9	0.05 ± 0.02	0.04 ± 0.01	0.06 ± 0.02	ns
C16:1 n7	0.06 ± 0.03 ^b^	0.13 ± 0.02 ^a^	0.15 ± 0.03 ^a^	***
C17:0	0.66 ± 0.03 ^b^	1.01 ± 0.02 ^b^	1.10 ± 0.02 c	***
C17:1	0.25 ± 0.02 ^c^	0.28 ± 0.01 ^b^	0.31 ± 0.02 ^a^	***
C18:0	11.74 ± 0.08 ^a^	11.45 ± 0.02 ^b^	11.19 ± 0.03 ^c^	***
C18:1 n9	22.60 ± 0.02 ^a^	21.95 ± 0.01 ^b^	21.33 ± 0.02 ^c^	***
C18:1 n7	0.03 ± 0.02	0.04 ± 0.02	0.03 ± 0.02	ns
C18:2 n6 (LA)	2.17 ± 0.03 ^c^	2.35 ± 0.03 ^b^	2.47 ± 0.03 ^a^	***
C18:3 n6	0.09 ± 0.01 ^c^	0.18 ± 0.02 ^b^	0.25 ± 0.01 ^a^	***
C18:3 n3 (ALA)	0.44 ± 0.01 ^c^	0.49 ± 0.03 ^b^	0.59 ± 0.01 ^a^	***
C20:0	0.25 ± 0.01 ^b^	0.27 ± 0.02 ^b^	0.32 ± 0.02 ^a^	***
C20:1 n9	2.38 ± 0.04 ^b^	2.47 ± 0.03 ^a^	2.49 ± 0.02 ^a^	***
C20:2 n6	0.12 ± 0.02 ^a^	0.09 ± 0.02 ^b^	0.10 ± 0.01 ^ab^	*
C20:3 n6	0.12 ± 0.02 ^a^	0.17 ± 0.02 ^a^	0.19 ± 0.02 ^a^	***
C20:4 n6	0.16 ± 0.05	0.15 ± 0.02	0.12 ± 0.02	ns
C20:3 n3	0.01 ± 0.00 ^b^	0.03 ± 0.01 ^a^	0.02 ± 0.00 ^b^	*
C20:5 n3	0.04 ± 0.01	0.03 ± 0.01	0.04 ± 0.01	ns
C22:0	0.03 ± 0.02 ^ab^	0.02 ± 0.01 ^b^	0.04 ± 0.01 ^a^	*
C22:1 n9	0.03 ± 0.02	0.04 ± 0.02	0.03 ± 0.01	ns
C22:2	0.10 ± 0.02	0.10 ± 0.03	0.14 ± 0.01	ns
C22:5 n6	0.05 ± 0.01	0.04 ± 0.02	0.04 ± 0.01	ns
C22:5 n3	0.07 ± 0.03 ^a^	0.06 ± 0.02 ^a^	0.02 ± 0.01 ^b^	*
C24:0	0.09 ± 0.02 ^c^	0.13 ± 0.02 ^b^	0.16 ± 0.01 ^a^	***
C22:6 n3	0.02 ± 0.01	0.03 ± 0.01	0.02 ± 0.01	ns
C24:1 n9	0.13 ± 0.01	0.13 ± 0.02	0.10 ± 0.01	ns
∑SFA	70.54 ± 0.45	70.61 ± 0.26	70.79 ± 0.46	ns
∑MUFA	26.03 ± 0.12 ^a^	25.69 ± 0.08 ^b^	25.26 ± 0.06 ^c^	***
∑PUFA	3.42 ± 0.14 ^c^	3.74 ± 0.13 ^b^	4.00 ± 0.01 ^a^	***
∑ n6/n3	4.60 ± 0.09 ^a^	4.70 ± 0.05 ^b^	4.60 ± 0.03 ^a^	***
AI	3.19 ± 0.01 ^a^	3.27 ± 0.01 ^b^	3.32 ± 0.01 ^c^	***
TI	4.31 ± 0.06 ^a^	4.36 ± 0.02 ^b^	4.38 ± 0.02 ^b^	***

A different letter corresponds to the significance of the data. *p* ≤ 0.05 = *; *p* ≤ 0.001 = ***. SFAs = saturated fatty acids; MUFAs = monounsaturated fatty acids; PUFAs = polyunsaturated fatty acids; LA = linoleic acid; ALA = alpha-linolenic acid. ns = not significance.

## Data Availability

The original contributions presented in the study are included in the article. Further inquiries can be directed to the corresponding author.
